# Accelerated Biological Age In Multiple Organs Is Associated With Incident Dementia

**DOI:** 10.1093/geroni/igaf122.161

**Published:** 2025-12-31

**Authors:** Fangyu Liu, Aditya Surapaneni, Jingsha Chen, Hamilton Se-Hwee Oh, Josef Coresh, Morgan Grams, Keenan Walker

**Affiliations:** Rutgers University, New Brunswick, New Jersey, United States; New York University, New York, New York, United States; Johns Hopkins Bloomberg School of Public Health, Baltimore, Maryland, United States; Icahn School of Medicine at Mount Sinai, New York, New York, United States; New York University, New York, New York, United States; New York University, New York, New York, United States; National Institute on Aging, Baltimore, Maryland, United States

## Abstract

Plasma-protein based biological age has been previously examined with respect to dementia risk at individual organ level. However, less is known about how accelerated aging in multiple organs may influence dementia risk. We estimated biological age in 11 organs using plasma proteomic data from the Atherosclerosis Risk in Communities (ARIC) study for participants in midlife (N = 11,595, mean age 57±6 years) and in late life (N = 4,287, mean age 75±5 years). When examined individually, we found that accelerated age in artery, brain, heart, immune, intestine, and liver during midlife (HR = 1.02-1.07, q < 0.05) was associated with higher 20-year dementia risk, after adjusting for demographic factors, BMI, eGFR, smoking status, APOE, diabetes, and hypertension. Accelerated age in all organs except kidney during late life (HR = 1.03-1.09, q < 0.05) was individually associated with higher 7-year dementia risk. When we examined the number of organs (0, 1, 2, or 3+) with abnormally old age (>1.5 SD), we observed that increasing number of abnormally old organs exhibited supra-additive associations with dementia risk in midlife and late life, albeit not statistically significant (p > 0.20). This finding suggests that the biological age of multiple systems may have synergistic effects on dementia development. Lastly, compared to participants who only had abnormally old brain age in midlife, participants with abnormally old age in both brain and the heart (HR = 2.82, p = 0.03) or both brain and muscle (HR = 2.54, p = 0.03) had higher 20-year dementia risk, suggesting a link between body and brain health. Our study provides new insights into the interactions of organ systems on dementia.

